# Body fat has stronger associations with bone mass density than body mass index in metabolically healthy obesity

**DOI:** 10.1371/journal.pone.0206812

**Published:** 2018-11-08

**Authors:** Yuan-Yuei Chen, Wen-Hui Fang, Chung-Ching Wang, Tung-Wei Kao, Yaw-Wen Chang, Chen-Jung Wu, Yi-Chao Zhou, Yu-Shan Sun, Wei-Liang Chen

**Affiliations:** 1 Department of Internal Medicine, Tri-Service General Hospital Songshan Branch, Tri-Service General Hospital; and School of Medicine, National Defense Medical Center, Taipei, Taiwan, Republic of China; 2 Division of Family Medicine, Department of Family and Community Medicine, Tri-Service General Hospital; and School of Medicine, National Defense Medical Center, Taipei, Taiwan, Republic of China; 3 Division of Geriatric Medicine, Department of Family and Community Medicine, Tri-Service General Hospital; and School of Medicine, National Defense Medical Center, Taipei, Taiwan, Republic of China; 4 Graduate Institute of Clinical Medical, College of Medicine, National Taiwan University, Taipei, Taiwan, Republic of China; 5 Division of Family Medicine, Department of Community Medicine, Taoyuan Armed Forces General Hospital, Taoyuan, Taiwan, Republic of China; University College London, UNITED KINGDOM

## Abstract

**Objective:**

The effect of obesity-induced metabolic abnormalities on bone mineral density (BMD) and osteoporosis are well established. However, the association between metabolically healthy obesity (MHO) and BMD remains unclear. Our aim was to investigate whether different obesity phenotypes in MHO were associated with BMD in a cross-sectional study.

**Methods:**

All eligible adults receiving a health examination at the Tri-Service General Hospital from 2010 to 2016 were included. They were categorized based on body mass index (BMI) or percentage body fat (PBF). The associations between BMI or PBF and BMD were analyzed by adjusting for pertinent covariables.

**Results:**

Males with normal weight and overweight and females with underweight and normal weight were associated with reduced BMD (***β*** = 0.221, 95%CI = -0.354, -0.088; ***β*** = -0.155, 95%CI = -0.286, -0.023) (***β* =** -0.736, 95%CI = -1.043, 0.429; ***β* =** -0.340, 95%CI = -0.567, -0.112), respectively. Females in Q1 had close to significant associations with reduced BMD (***β* =** -0.253, 95%CI = -0.465, -0.041). Normal weight, overweight, Q2, and Q3 had stronger prediction of low BMD with ORs of 0.402 (95%CI = 0.204–0.791), 0.539 (95%CI = 0.321–0.905), 0.694 (95%CI = 0.490–0.982), and 0.466 (95%CI = 0.342–0.636), respectively. The relationship remained significant in male population that PBF was associated with reduced BMD with ORs of 0.435 (95%CI = 0.203, 0.935), 0.494 (95%CI = 0.247, 0.991), 0.268 (95%CI = 0.120, 0.597) in Q1, Q2, Q3 respectively.

**Conclusion:**

Increased PBF had a significant association with low BMD in the MHO population. Obesity defined by PBF might be a useful indicator for low BMD. The association between body fat and bone health deserves further investigation regarding the potential pathophysiological mechanisms.

## Introduction

Osteoporosis, diagnosed by measurement of bone mineral density (BMD), is a common health problem worldwide due to its high risk for fractures, morbidity and mortality and expensive health care costs[1]. Impacts on BMD are caused by a multifactorial etiology, including metabolic syndrome (MetS) and obesity. Accumulated evidence indicates significant associations between osteoporosis and metabolic abnormalities such as central obesity, hypertension, hyperglycemia and dyslipidemia [2–4]. It is well established that low body mass is correlated with reduced BMD and an increased risk of osteoporosis. However, controversial studies have indicated that increased percentage body fat (PBF) and abdominal fat accumulation are strongly associated with low BMD[5, 6].

Sarcopenic obesity, defined as low muscle mass and the presence of obesity, has been reported to be associated with an increased risk of osteoporosis and non-vertebral fracture[7]. Sarcopenic obesity has also been suggested to be associated with low BMD in the elderly population[8]. Metabolically healthy obesity (MHO), a condition observed in a subgroup of obese individuals who do not display MetS, was recently reported in several studies[9, 10]. People with MHO are prevalent and are generally agreed to comprise up to 25%, but not more than 30%, of the adult obese population[11, 12]. It was proposed that those with MHO had lower risks of cardiovascular diseases and preserved insulin sensitivity[13]. However, unlike the relationship of obesity and BMD, the potential association between MHO and BMD is largely unexplored. The aim of our study was to investigate whether having MHO individuals was associated with BMD in a cross-sectional study composed of participants receiving a health examination at the Tri-Service General Hospital (TSGH) in Taiwan.

## Methods

### Study design

This cross-sectional study was composed of male and female participants aged 20 years old and older who underwent comprehensive health examinations in the TSGH. Study approval was given by the Institutional Review Board of Tri-Service General Hospital, Taiwan. The Institutional Review Board waived the requirement for individual informed consent because the data were analyzed anonymously. MHO is defined as a specific feature of obesity that is not accompanied by MetS. Exclusion criteria of our study were chronic liver diseases, inflammatory bowel disease, chronic kidney disease, cancer, lupus, multiple myeloma, rheumatoid arthritis, thyroid disorders, and missing information (including baseline characteristics, and dual energy x-ray absorptiometry (DEXA)) (N = 56178). Additionally, those with MetS as defined by the Taiwan Health Promotion Administration of the Ministry of Health and Welfare in 2007 were also excluded (N = 6269).[14]. In the final analysis, there were 6776 metabolically healthy participants. For body mass index (BMI), we divided all eligible subjects into 4 categories: (1) underweight: BMI<18.5 (N = 380); (2) normal weight: 18.5<BMI<24 (N = 3747); (3) overweight: 24<BMI<27 (N = 1841); and (4) obese: BMI>27 (N = 797). Obesity was defined as a BMI> 27 kg/m^2^ according to the criteria of the Department of Health in Taiwan[15]. For PBF, the study sample was divided again into 4 categories by classifying the PBF values of subjects in quartiles: (1) Q1: PBF<22.8 (N = 1565); (2) Q2: 22.8<PBF<27.2 (N = 1541); (3) Q3: 27.2<PBF<32.4 (N = 1522); and (4) Q4: PBF>32 (N = 1532).

### Measurement of BMD

DEXA, the most frequently used technique for measuring BMD, was performed during the health examinations by using a Prodigy Series X-Ray Tube Housing Assembly (GE Medical Systems Lunar 3030 Ohmeda Dr Madison, Wisconsin, USA). DEXA can be applied to measure BMD at various body sites. The density of the lumbar spine was measured rather than the total hip. We excluded those participants with past histories of vertebral fracture, vertebroplasty, or implants of polymethylmethacrylate cement.

### Diagnosis of low BMD and osteoporosis

Lumbar spine osteoporosis and low BMD were defined in the health examinations based on the criteria of the World Health Organization[16]. The definition of osteoporosis was a BMD less than or equal to 2.5 SDs below that of a young, healthy adult female reference group [17]. Low BMD was diagnosed when the lumbar spine BMD value was between -1 and -2.5 SDs below that of the young reference group.

### Measurement of PBF

PBF was the indicator used in the study and was measured by BIA (InBody720, Biospace, Inc., Cerritos, CA, USA), which was non-invasive, portable, and inexpensive. This accurate technique is effective and has been validated to estimate body composition parameters such as total body weight, extracellular water, intracellular water, and fat mass[18].

### Definition of MetS

MetS was diagnosed if an individual had ≧3 of the following characteristics based on the Taiwan Health Promotion Administration of the Ministry of Health and Welfare in 2007[14]: (1) waist circumference (WC): male>90 cm and female>80 cm; (2) systolic blood pressure (SBP)≧130 mmHg or diastolic blood pressure (DBP)≧80 mmHg, or self-reported hypertension; (3) triglyceride (TG)≧150 mg/dL (1.7 mmol/L); (4) fasting plasma glucose (FPG)≧100 mg/dL, or a past history of diabetes status; and (5) high density lipoprotein cholesterol (HDL-C): male<40 mg/dL (1.03 mmol/L) and female<50 mg/dL (1.3 mmol/L).

### Covariate measurements

BMI was estimated based on a general formula where the weight of the individual in kilograms was divided by the square of the individual’s height in meters (kg/m^2^). Biochemical data were collected by drawing blood samples from subjects after fasting for at least 8 hours; the samples were measured by standard procedures. Data on cigarette smoking were obtained from participants by asking the question “How many packs do you smoke per day”. Consumption of alcohol was determined by self-report questionnaire, and participants were divided into ‘‘never” and ‘‘alcohol consumption” groups. Proteinuria was measured in a random urine sample during the health examination and in a first morning void urine sample collected by the participant. Proteinuria was diagnosed by dipstick test, which is a basic diagnostic tool for determining pathological changes in urine sample in standard urinalysis[19].

### Statistical analysis

Statistical estimations used in the study were performed using the Statistical Package for the Social Sciences, version18.0 (SPSS Inc., Chicago, IL, USA) for Windows. The differences between males and females in terms of demographic information and biochemistry data were examined by Student’s t and Pearson's chi-square tests. A two-sided *p*-value of ≤ 0.05 was regarded as the threshold for statistical significance. An extend-model approach was used with multivariable adjustment for pertinent clinical variables as follows: Model 1 = age + gender; Model 2 = Model 1 + proteinuria, total cholesterol (TC), uric acid (UA), creatinine (Cr), aspartate transaminase (AST), albumin, high sensitivity C-reactive protein (hsCRP), and thyroid stimulating hormone (TSH); and Model 3 = Model 2 + history of cigarette smoking and alcohol consumption. Natural logarithm transformation was performed to normalize the distributions of all variables before analysis because variables were nonlinearly related to the response variable. Linear regression was performed to assess the association between different obesity phenotypes and BMD. Logistic regression was used to investigate gender differences in the associations between different obesity phenotypes and the presence of low BMD and osteoporosis.

## Results

### Epidemiological characteristics

Table 1 shows the characteristics of participants categorized by BMI (underweight: BMI<18.5; normal weight: 18.5<BMI<24; overweight: 24<BMI<27; obese: BMI>27) or PBF (Q1: PBF<22.8; Q2: 22.8<PBF<27.2; Q3: 27.2<PBF<32.4; Q4: PBF>32.4). The mean ages of the underweight, normal weight, overweight, and obese BMI subgroups were 42.48±13.55, 48.00±12.41, 51.01±11.95, and 49.00±12.47 years, respectively. The mean ages of the Q1, Q2, Q3, and Q4 PBF subgroups were 46.80±12.62, 49.48±12.00, 49.44±11.74, and 52.18±12.48 years, respectively. The percentages of males in each BMI subgroup were 21.2% (underweight), 44.6% (normal weight), 72.7% (overweight), and 72.5% (obese). In the PBF subgroups, the percentages of males were Q1: 85.4%, Q2: 67.5%, Q3: 40.3%, and Q4: 17.1%. All continuous variables, smoking history and alcohol consumption were significantly different among the BMI subgroups (P<0.05). By contrast, all continuous variables except AST, and smoking history and alcohol consumption were significantly different among the PBF subgroups (P<0.05).

**Table 1:** Characteristics of study sample.

	**BMI**				
**Variables**	**BMI<18.5** **Underweight**	**18.5<BMI<24** **Normal weight**	**24<BMI<27** **Overweight**	**BMI>27** **Obese**	P Value
**Continuous Variables, median (IQR)**					
**BMD**	-0.30 (1.60)	-0.20 (1.70)	0.60 (1.90)	0.80 (1.70)	<0.001
**Age**	45.27 (22.58)	50.72 (17.35)	52.92 (16.09)	51.12 (18.09)	<0.001
**TC**	184.00 (43.00)	189.00 (48.00)	190.00 (47.00)	191.00 (49.00)	<0.001
**UA**	4.30 (1.30)	5.10 (1.80)	6.10 (1.80)	6.40 (2.10)	<0.001
**Cr**	0.70 (0.00)	0.80 (0.00)	0.90 (0.00)	0.90 (0.00)	<0.001
**AST**	18.00 (7.00)	19.00 (7.00)	20.00 (7.00)	21.00 (9.00)	<0.001
**Albumin**	4.50 (0.00)	4.50 (0.00)	4.50 (0.00)	4.50 (0.00)	<0.001
**hsCRP**	0.04 (0.00)	0.07 (0.00)	0.11 (0.00)	0.20 (0.00)	<0.001
**TSH**	2.07 (2.00)	1.98 (1.00)	1.86 (1.00)	1.99 (2.00)	0.003
**Category Variables, (%)**					
**Gender (male)**	74 (21.2)	1478 (44.6)	1136 (72.7)	451 (72.5)	<0.001
**Proteinuria**	127 (37.5)	1201 (35.4)	540 (31.9)	270 (35.9)	0.202
**Smoking**	82 (21.6)	1033 (27.6)	629 (34.3)	297 (37.5)	<0.001
**Drinking**	117 (38.4)	1427 (46.1)	846 (55.8)	359 (55.9)	<0.001
	**PBF**				
**Variables**	**PBF<22.8** **Q1**	**22.8<PBF<27.2** **Q2**	**27.2<PBF<32.4** **Q3**	**PBF>32.4** **Q4**	P Value
**Continuous Variables, median (IQR)**					
**BMD**	0.50 (1.80)	0.40 (1.80)	0.40 (1.70)	0.20 (2.00)	<0.001
**Age**	48.56 (18.72)	51.12 (16.98)	51.05 (16.03)	53.26 (16.32)	<0.001
**TC**	184.00 (46.00)	191 (50.00)	188.00 (45.00)	196.00 (47.00)	<0.001
**UA**	5.80 (1.60)	5.80 (2.20)	5.10 (2.20)	5.20 (1.80)	<0.001
**Cr**	0.90 (0.00)	0.90 (0.00)	0.70 (0.00)	0.70 (0.00)	<0.001
**AST**	19.00 (7.00)	19.00 (8.00)	19.00 (7.00)	19.00 (8.00)	0.114
**Albumin**	4.50 (0.00)	4.50 (0.00)	4.50 (0.00)	4.40 (0.00)	<0.001
**hsCRP**	0.06 (0.00)	0.08 (0.00)	0.09 (0.00)	0.15 (0.00)	<0.001
**TSH**	1.85 (1.00)	1.90 (1.00)	1.96 (1.00)	2.14 (2.00)	<0.001
**Category Variables, (%)**					
**Gender (male)**	1173 (85.4)	891 (67.5)	522 (40.3)	215 (17.1)	<0.001
**Proteinuria**	528 (37.3)	492 (35.1)	500 (35.9)	523 (36.9)	0.927
**Smoking**	709 (45.4)	565 (36.8)	396 (26.1)	228 (14.9)	<0.001
**Drinking**	846 (62.0)	777 (56.2)	620 (45.4)	465 (33.9)	<0.001

IQR, inter quartile range; BMD, bone mineral density; TC, total cholesterol; UA, uric acid; Cr, creatinine; AST, aspartate transaminase; hsCRP, high sensitive C-reactive protein; TSH, thyroid stimulating hormone.

### Associations between different obesity phenotypes and BMD

Table 2 shows the associations between different obesity phenotypes and BMD by gender. In males, being of normal weight and being overweight were associated with reduced BMD with ***β*** values of -0.221 (95%CI = -0.354, -0.088) and -0.155 (95%CI = -0.286, -0.023), respectively. However, no significant difference was observed between PBF and BMD. In the female population, being of underweight and being normal weight were significantly associated with reduced BMD with ***β*** values of -0.736 (95%CI = -1.043, 0.429) and -0.340 (95%CI = -0.567, -0.112), respectively. Being in Q1 was related to reduced BMD with a ***β*** value of -0.253 (95%CI = -0.465, -0.041).

**Table 2:** Association between different obesity phenotypes and BMD in gender difference.

Gender	BMI/PBF groups		Model ^a^ 1 *β*^b^ (95% CI)	*P* Value	Model ^a^ 2 *β*^b^ (95% CI)	*P* Value	Model ^a^ 3 *β*^b^ (95% CI)	*P* Value
			BMD					
**Male**	**BMI**	**Underweight vs Obese**	-0.658 (-1.256, -0.061)	0.031	-0.552 (-1.156, 0.051)	0.073	-0.560 (-1.163, 0.044)	0.069
		**Normal weight vs Obese**	-0.246 (-0.376, -0.116)	<0.001	-0.222 (-0.355, -0.089)	<0.001	-0.221 (-0.354, -0.088)	<0.001
		**Overweight vs Obese**	-0.167 (-0.299, -0.036)	0.012	-0.153 (-0.285, -0.022)	0.022	-0.155 (-0.286, -0.023)	0.023
	**PBF**	**Q1 vs Q4**	0.033 (-0.137, 0.204)	0.701	0.053 (-0.121, 0.227)	0.551	0.062 (-0.113, 0.236)	0.489
		**Q2 vs Q4**	0.069 (-0.103, 0.242)	0.429	0.084 (-0.089, 0.257)	0.343	0.088 (-0.085, 0.261)	0.318
		**Q3 vs Q4**	0.059 (-0.122, 0.241)	0.523	0.077 (-0.105, 0.259)	0.405	0.083 (-0.099, 0.265)	0.370
**female**	**BMI**	**Underweight vs Obese**	-0.747 (-1.047, -0.447)	<0.001	-0.736 (-1.043, -0.429)	<0.001	-0.736 (-1.043, 0.429)	<0.001
		**Normal weight vs Obese**	-0.350 (-0.572, -0.127)	0.002	-0.345 (-0.572, -0.117)	0.003	-0.340 (-0.567, -0.112)	0.003
		**Overweight vs Obese**	-0.035 (-0.287, 0.216)	0.782	-0.026 (-0.279, 0.227)	0.838	-0.025 (-0.279, 0.228)	0.845
	**PBF**	**Q1 vs Q4**	-0.211 (-0.417, -0.005)	0.045	-0.255 (-0.466, -0.044)	0.018	-0.253 (-0.465, -0.041)	0.019
		**Q2 vs Q4**	-0.125 (-0.287, 0.036)	0.127	-0.140 (-0.306, 0.026)	0.098	-0.131 (-0.297, 0.035)	0.122
		**Q3 vs Q4**	-0.106 (-0.237, 0.025)	0.112	-0.105 (-0.239, 0.028)	0.122	-0.103 (-0.236, 0.031)	0.132

^a^ Adjusted covariates:

Model 1 = age

Model 2 = Model 1 + proteinuria, TC, UA, Cr, AST, albumin, hsCRP, TSH

Model 3 = Model 2 + history of smoking, drinking

***β***^**b**^ was interpreted as change of BMD for each increase in obesity phenotypes

### Associations between different obesity phenotypes and the presence of low BMD and osteoporosis

Table 3 shows odd ratios (ORs) of different obesity phenotypes for predicting the presence of low BMD and osteoporosis as obtained by multivariable logistic regression. Being of normal weight and overweight could predict the presence of low BMD with ORs of 0.402 (95%CI = 0.204, 0.791) and 0.539 (95%CI = 0.321, 0.905), respectively. Being in Q2 and Q3 were associated with low BMD with ORs of 0.694 (95%CI = 0.490, 0.982) and 0.466 (95%CI = 0.342, 0.636), respectively, in the fully adjusted model. However, no significant differences were noted among the associations with osteoporosis in the study sample.

**Table 3:** Association between different obesity phenotypes with the presence of low BMD and osteoporosis.

	Variable	Model ^a^ 1 OR (95% CI)	*P* Value	Model ^a^ 2 OR (95% CI)	*P* Value	Model ^a^ 3 OR (95% CI)	*P* Value
**Low BMD**							
**BMI**	**Obese**	Reference	-	Reference	-	Reference	-
	**Overweight**	1.358 (0.889–2.704)	0.157	0.533 (0.318–0.895)	0.017	0.539 (0.321–0.905)	0.019
	**Normal weight**	2.310 (1.566–3.408)	<0.001	0.399 (0.203–0.785)	0.008	0.402 (0.204–0.791)	0.008
	**Underweight**	5.064 (3.168–8.097)	<0.001	0.530 (0.202–1.389)	0.196	0.533 (0.203–1.399)	0.201
**PBF**	**Q4**	Reference	-	Reference	-	Reference	-
	**Q3**	0.511 (0.395–0.661)	<0.001	0.466 (0.342–0.636)	<0.001	0.466 (0.342–0.636)	<0.001
	**Q2**	0.606 (0.473–0.775)	<0.001	0.693 (0.490–0.980)	0.038	0.694 (0.490–0.982)	0.039
	**Q1**	0.629 (0.493–0.803)	<0.001	0.764 (0.500–1.168)	0.215	0.761 (0.498–1.163)	0.207
**Osteoporosis**							
**BMI**	**Obese**	Reference	-	Reference	-	Reference	-
	**Overweight**	2.132 (0.471–9.661)	0.326	0.441 (0.079–2.454)	0.350	0.450 (0.081–2.499)	0.361
	**Normal weight**	3.427 (0.823–14.271)	0.091	0.126 (0.015–1.083)	0.059	0.124 (0.014–1.070)	0.058
	**Underweight**	16.523 (3.782–72.182)	<0.001	0.185 (0.012–2.915)	0.230	0.179 (0.011–2.823)	0.221
**PBF**	**Q4**	Reference	-	Reference	-	Reference	-
	**Q3**	0.561 (0.288–1.091)	0.088	0.555 (0.255–1.208)	0.138	0.562 (0.257–1.226)	0.148
	**Q2**	0.433 (0.211–0.888)	0.022	0.623 (0.248–1.564)	0.313	0.623 (0.247–1.573)	0.317
	**Q1**	0.709 (0.382–1.315)	0.275	1.109 (0.391–3.144)	0.846	1.122 (0.395–3.192)	0.829

^a^ Adjusted covariates:

Model 1 = age

Model 2 = Model 1 + proteinuria, TC, UA, Cr, AST, albumin, hsCRP, TSH

Model 3 = Model 2 + history of smoking, drinking

The results based on gender differences are demonstrated in Table 4. There were no significant findings regarding different obesity phenotypes and low BMD in the female population. On the contrary, male subjects displayed the same results as described above. ORs were 0.279 (95%CI = 0.104, 0.750) and 0.360 (95%CI = 0.170, 0.760) in the normal weight and overweight groups, respectively, and 0.435 (95%CI = 0.203, 0.935), 0.494 (95%CI = 0.247, 0.991), and 0.268 (95%CI = 0.120, 0.597) in Q1, Q2, and Q3, respectively.

**Table 4:** Association between different obesity phenotypes with the presence of low BMD in sex difference.

Sex	Variables	Model ^a^ 1 OR (95% CI)	*P* Value	Model ^a^ 2 OR (95% CI)	*P* Value	Model ^a^ 3 OR (95% CI)	*P* Value
	Low BMD						
**Male**							
**BMI**	**Obese**	Reference	-	Reference	-	Reference	-
	**Overweight**	1.309 (0.693–2.473)	0.407	0.361 (0.171–0.762)	0.008	0.360 (0.170–0.760)	0.007
	**Normal weight**	3.127 (1.739–5.623)	<0.001	0.282 (0.105–0.757)	0.012	0.279 (0.104–0.750)	0.011
	**Underweight**	13.656 (6.173–30.213)	<0.001	0.304 (0.066–1.405)	0.127	0.294 (0.063–1.366)	0.118
**PBF**	**Q4**	Reference	-	Reference	-	Reference	-
	**Q3**	0.506 (0.238–1.076)	0.077	0.271 (0.122–0.604)	<0.001	0.268 (0.120–0.597)	<0.001
	**Q2**	1.229 (0.659–2.291)	0.514	0.498 (0.249–0.997)	0.049	0.494 (0.247–0.991)	0.047
	**Q1**	1.763 (0.967–3.213)	0.064	0.437 (0.204–0.938)	0.034	0.435 (0.203–0.935)	0.033
**Female**							
**BMI**	**Obese**	Reference	-	Reference	-	Reference	-
	**Overweight**	1.314 (0.729–2.370)	0.364	0.735 (0.338–1.599)	0.438	0.763 (0.351–1.661)	0.496
	**Normal weight**	1.125 (0.657–1.928)	0.668	0.420 (0.154–1.143)	0.090	0.436 (0.160–1.188)	0.104
	**Underweight**	1.797 (0.969–3.333)	0.063	0.657 (0.168–2.566)	0.545	0.686 (0.175–2.686)	0.589
**PBF**	**Q4**	Reference	-	Reference	-	Reference	-
	**Q3**	0.670 (0.506–0.888)	0.005	0.695 (0.478–1.010)	0.057	0.694 (0.477–1.009)	0.056
	**Q2**	0.904 (0.655–1.247)	0.537	1.123 (0.702–1.797)	0.629	1.122 (0.701–1.797)	0.631
	**Q1**	0.680 (0.429–1.076)	0.099	0.671 (0.351–1.285)	0.229	0.654 (0.342–1.253)	0.201

^a^ Adjusted covariates:

Model 1 = age

Model 2 = Model 1 + proteinuria, TC, UA, Cr, AST, albumin, hsCRP, TSH

Model 3 = Model 2 + history of smoking, drinking

## Discussion

In the present study, we highlighted the associations between different obesity phenotypes and BMD in an MHO population from a large population-based survey. We observed that obesity as defined by BMI was closely associated with increased BMD, and that obesity as defined by PBF was related to reduced BMD. Not only BMI but also PBF had a likelihood of predicting the presence of low BMD, particularly in the male population. To the best of our knowledge, our study is the first to explore the associations between different obesity phenotypes and low BMD and osteoporosis in an adult population.

Controversial findings have been reported in several studies on the impact of obesity on bone metabolism. Obesity has conventionally been suggested to be beneficial to bones and protective against osteoporosis[20]. Salamat et al. reported that obesity, defined by BMI, conferred a reduced risk for osteoporosis and low BMD in a non-institutionalized population[21]. Body fat and lean mass were suggested to contribute to the maintenance of BMD, by generating a mechanical overload on the bones[22, 23]. However, recent evidence has shown that excess body fat might not have a beneficial effect on BMD[24]. Sarcopenic obesity, a specific term for the presence of decreased muscle mass and increased body fat, was reported to be associated with the development of osteoporosis among an elderly population[7, 8]. A similar result was presented by Zhao et al., who reported that fat mass was inversely correlated with bone mass genetically, environmentally, and phenotypically[25]. In a Korean study, BMI was considered as a protective factor against vertebral fractures and was related to increased BMD; however, PBF was a risk factor for vertebral fractures and low BMD[26]. A recent study with Pacific Island women showed that PBF was inversely associated with BMD[27]. Accumulated visceral adipose tissue was associated with low BMD in middle-aged Chinese women[28]. This is consistent with our findings that increased PBF values had a harmful effect on bone health and could predict the risk of developing low BMD. Although positive relationship with BMD has been reported in previous studies, higher BMI had a tendency to predict a risk of lower BMD in our study. The underlying mechanism for this observation was unclear. In a cross-sectional study including women of different ethnicities, higher BMI caused increased BMD for white women while it reduced BMD in African Americans[29]. It appears that there is a race-dependent effect of obesity on BMD.

Several experimental and clinical studies have suggested that obesity is detrimental to bone health. Proinflammatory cytokines such as TNF-α, IL-1, and IL-6, which are induced by adipose tissues, contribute to the development of osteoclast activity and bone resorption[30]. The stimulating mechanism of osteoclasts was via the regulation of RANKL/RANK/OPG[31]. Adipogenesis might be a plausible pathway for the impact of obesity on reduced bone formation, because adipocytes and osteoblasts originate from common multi-potential mesenchymal stem cells[32]. In obese animal models, altered bone metabolism might result from the overproduction of leptin[33]. Increased secretion of leptin or decreased production of adiponectin could result in macrophages accumulation induced by adipocytes[34].

A gender difference in the association between obesity with BMD was observed in our study based on our finding that only among male subjects did obesity have tendency for predicting the presence of low BMD. Because the study sample of health examinations was derived from an adult population, most female individuals had not experienced the dramatic drop in circulating estradiol levels that accompanies menopause. A previous study reported that significant changes of hormones in women were believed to be the major cause of rapid bone loss in women during menopause[35]. Estrogen inhibited bone resorption by inducing cumulative changes in multiple estrogen-dependent regulatory factors to affect osteoclast formation[36]. The protective role of sex hormones appeared to be a plausible explanation for the varying results regarding BMD by gender.

There were several limitations to the present study. First, the sample analyzed in our study was composed of a relatively healthy general adult population. Participants with osteoporosis were rare and not prevalent in this age group. It was not surprising that no significant differences were observed in the associations between obesity phenotypes and the presence of osteoporosis. Second, the dataset was derived from an exclusively Asian population, so the limited ethnic diversity of the participants might not reflect the racial differences that exist for the association of obesity with BMD. Next, this was a cross-sectional design, so causal inferences are no not able to be made; a longitudinal survey is suggested for use in further studies. Finally, various factors can limit the use of BIA. Relative increases in extracellular water and total body water might underestimate the percentage of body fat and overestimate fat-free mass in the obese state[18]. Food intake can lower the results of body fat measurements by causing a variation between the highest and lowest PBF readings of PBF[37]. Overestimation of fat mass and underestimation of PBF are observed after moderate exercise due to reduced impedance[38].

## Conclusion

Our findings demonstrated that increased PBF was significantly associated with reduced BMD after adjusting for variable confounders in an MHO sample and that high PBF might be a useful indicator for low BMD. A gender difference was noted, indicating that hormones might be a key factor influencing bone metabolism. The association between body fat and bone health deserves further investigation into the potential pathophysiological mechanisms. Strategies for preventing the detrimental impact of obesity and prolonged follow-up research for predicting risks of incident low BMD and even osteoporosis are necessary.
